# Drug Poisoning Deaths according to Ethnicity in Utah

**DOI:** 10.1155/2013/380161

**Published:** 2013-12-11

**Authors:** Ray M. Merrill, Riley J. Hedin, Anna Fondario, Arielle A. Sloan, Carl L. Hanson

**Affiliations:** ^1^Department of Health Science, Brigham Young University, Provo, UT 84602, USA; ^2^Violence and Injury Prevention Program, Utah Department of Health, Salt Lake City, UT 84114, USA

## Abstract

This study characterizes drug-related deaths according to ethnicity in Utah during 2005–2010, based on data from the Utah Violent Death Reporting System (UTVDRS). Hispanics made up 12.1% (12.5% male and 11.7% female) of deaths. The most frequently identified drugs among decedents were opiates, then illicit drugs, benzodiazepines, over-the-counter medication, and antidepressants. Death rates for each drug were significantly greater in non-Hispanics than Hispanics. Most decedents used a combination of drugs. For each combination, rates were significantly greater for non-Hispanics than Hispanics, with an exception for opiates and illicit drugs combined, where there was no significant difference. Approximately 79% of non-Hispanics and 65% of Hispanics had one or more of the selected problems (e.g., mental, physical, or crisis related). Rates for each combination of problems were significantly greater in non-Hispanics, with the exception of crisis. Hispanics were less affected by the rise in prescription drug abuse. Hispanic decedents had a greater proportion of illegal drugs, consistent with it being more difficult to obtain prescription drugs. Hispanic decedents were less likely to have physical and mental health problems, which may be related to a smaller chance of diagnosis of such problems through the healthcare system.

## 1. Introduction

Drug-related death rates in the United States have steadily increased in the past decade. Deaths from drug poisonings more than doubled from 6 per 100,000 in 2000 to 12.5 per 100,000 in 2010 [1]. Since 2009, deaths due to drug poisonings have exceeded deaths from motor vehicle accidents, with males being at greater risk than females, and death rates peaking at age of 45–54 [1]. Several studies have reported increasing death rates from opioid analgesics (e.g., oxycodone, methadone, or hydrocodone) [2–7]. Since 2003, more overdose deaths have resulted from opioid analgesics than heroin and cocaine combined [6].

In 2010, the drug poisoning rate in Utah was 16.9 per 100,000, compared with 12.3 per 100,000 in the United States [8]. Utah ranked eighth highest in the nation. Recent studies have explored selected aspects of prescription opioid-related deaths in Utah [9, 10]. However, these studies have not looked at the influence of ethnicity on drug-related deaths. It has previously been observed that racial/ethnic minorities in the United States are less likely to use prescription drugs and, consequently, less likely to abuse them [11]. In the current study, we examine whether drug-related death rates are lower for Hispanics than non-Hispanics in Utah, according to age and gender. The study also explores whether differences exist in specific types of drugs implicated with the decedents and if problems experienced just prior to death differ between ethnicities. This information may help public health officials better understand and successfully intervene in reducing drug-related deaths among Hispanics and non-Hispanics.

## 2. Materials and Methods

A retrospective cohort study was conducted on drug overdose deaths in Utah. The Hispanic-Latino (hereafter Hispanic) population in Utah in 2012 represented 13.3% of the state's population [12]. In addition, the Hispanic population consisted of the following:

- 49.7% female.
- 31.2% under 18 years of age.
- 9.5% over 65 years old.
- 66.8% of adults (18 years and older) married.
- Median household income of $57,783 (during 2007–2011).
- 90.6% with a high school degree and 29.6% with a bachelor's degree or higher among those aged 25 years and older [12, 13].

All drug-related deaths identified for this study occurred in Utah from 2005 through 2010. Data were collected from death certificates, police reports, and reports produced by the Office of the Medical Examiner (OME) and entered into the Utah Violent Death Reporting System (UTVDRS) [14]. The OME investigates each sudden or unexpected death [15]. Police and medical examiner reports document the scene of death with pictures, detailed notes, interviews with witnesses or family members and friends of the victim, autopsies, and toxicology testing. State-level data are then pooled together in the National Violent Death Reporting System (NVDRS), a secure database implemented by the Centers for Disease Control and Prevention (CDC).

For each drug-related death in the current study, abstractors coded for selected variables, such as age, sex, ethnicity, history of mental illness, and alcohol or substance use/abuse, and also wrote a short narrative that summarized the investigative findings from medical examiner and law enforcement records. Accidental drug overdose deaths were incorporated into the UTVDRS system using the same coding manual and abstraction procedures of UTVDRS cases. To control for inconsistency among the various sources of data, abstractors participated in a coding training and a review of the coding manual. In addition, ongoing coding support was provided through the CDC, the UTVDRS team epidemiologist, and the lead abstractor. Further, the team epidemiologist reviewed each incident as it was being abstracted to check for coding errors, to ensure that endorsed circumstance variables were supported in the narrative, and to run logic queries on the data. Cases with discrepancies were flagged for further abstractor review. Further, data were analyzed by a hierarchical rule for each variable. The hierarchical rule used for our data is based on the rules set forth by the CDC. Data sources were ranked in terms of their potential reliability for each data element. For example, age of the victim was taken first from the death certificate, second from the medical examiner report, and finally from police records. When data sources had complete but discordant data, the hierarchical rule was also used for each variable. Some UTVDRS variables, including mental illness, relied on information from friends and family members when proper records were unavailable.

The process of compiling the drug overdose death data used in this study occurred during June through September 2011. There were 2,843 drug-related deaths in Utah from 2005 through 2010 (31.2% accidents, 13.3% suicides, and 55.5% undetermined intent for non-Hispanics and 25.3% accidents, 9.9% suicides, and 64.8% undetermined intent for Hispanics). Forty persons had unknown ethnicity and were therefore excluded from the current study. Institutional Review Board approval was obtained from the Utah Department of Health.

### 2.1. Description of Measures

The primary outcome measure was drug type. Drugs were classified as opioid analgesics (pain relievers), benzodiazepines (to relieve nervousness), antidepressants (to relieve or prevent psychic depression), illicit (e.g., cocaine, heroin, and methamphetamines), and over-the-counter.

Selected demographic variables were considered, wherein age was categorized as 0–24, 25–34, 35–44, 45–54, and 55 and older, and ethnicity was represented by Hispanics and non-Hispanics. The frequency of seven events that may have occurred just prior to the decedents drug overdose was assessed among the decedents: current mental health problem, current physical health problem, crisis in the past two weeks, alcohol problem, job problem, financial problem, and recent criminal legal problem. They were reported based on the medical examiner report and law enforcement narratives. Brief descriptions of these variables from the coding manual are as follows.

- Mental health problem: the victim was experiencing mental health problems that were relevant to the event.
- Physical health problem: the victim was experiencing physical health problems that were relevant to the event.
- Recent crisis: it identifies cases in which a very current crisis or acute precipitating event appears to have contributed to the death two weeks prior to or would have occurred within two weeks after the incident.
- Alcohol problem: the victim was perceived by self or others to have a problem with, or to be addicted to, alcohol. There does not need to be any indication that the problem directly contributed to the death.
- Job problem: it is coded as “yes” if, at the time of the incident, the victim was either experiencing a problem at work or was having a problem with joblessness, and this appears to have contributed to the death.
- Financial problem: it is coded as “yes” if, at the time of the incident, the victim was experiencing a problem such as bankruptcy, overwhelming debts, or foreclosure of a home or business, and this appears to have contributed to the death.
- Recent criminal legal problem: it is coded as “yes” if, at the time of the incident, the victim was facing criminal legal problems and this appears to have contributed to the death.

### 2.2. Statistical Techniques

Percentages and rates were used to describe the decedents. Rates were calculated with population estimates for Hispanics and non-Hispanics obtained from the U.S. Census Bureau [16]. Poisson regression was also used to assess associations, adjusting for age and sex. Distributions of age, sex, drug type, and selected problems were compared between Hispanics and non-Hispanics and tested for statistical significance using the chi-square test. Tests of significance were based on two-sided hypothesis tests using the 0.05 level. Analysis was performed using Statistical Analysis System (SAS) software version 9.3 (SAS Institute Inc., Cary, NC, USA, 2010).

## 3. Results

During 2005 through 2010, the percent of the Utah population that was Hispanic was 12.1 (12.5 for males and 11.7 for females). Among Hispanics, racial classifications were 92.1% White, 2.3% Black, 4.0% American Indian/Alaska Native, and 1.5% Asian or Pacific Islander. Corresponding classifications for non-Hispanics were 94.0%, 1.3%, 1.3%, and 3.5%. The percent of the Utah population who were Hispanic steadily increased during 2005 through 2010, from 11.5 to 13.4 in males and from 10.5 to 12.7 in females. The increasing percentages occurred across the following age groups: 13.1 to 16.0 (22.3%) in ages 0–24, 13.5 to 14.0 (3.3%) in ages 25–34, 11.9 to 15.0 (26.4%) in ages 35–44, 7.5 to 10.1 (34.8%) in ages 45–54, and 4.6 to 5.5 (20.6%) in ages 55 years and older.

Drug-related overdose deaths are presented according to ethnicity in Table 1. The distribution of males and females was not significantly different between non-Hispanic and Hispanic decedents (chi-square *P* = 0.8708). In addition, the age distribution did not significantly differ between the ethnic groups (chi-square *P* = 0.1641). Approximately 58% of decedents were male and about 75% were aged from 25 to 54 years. The death rate (per 100,000) attributed to drug poisoning was 19.2 for non-Hispanics compared with 7.4 for Hispanics (Rate ratio = 2.59, 95% CI = 2.18–3.06). The higher rate ratio observed in non-Hispanics was more pronounced in males than females and became insignificant in the oldest age group.

The most frequently identified drugs among decedents were opiates, followed by illicit drugs, benzodiazepines, over-the-counter medication, and finally antidepressants (Table 2). The distribution of the selected drug types shown in the table significantly differed between non-Hispanics and Hispanics (chi-square *P* = 0.0004). Non-Hispanic decedents reflected a greater percentage of opiates and a lower percentage of illicit drugs compared with Hispanics. Rates of death associated with each drug type were significantly greater in non-Hispanics than in Hispanics.

**Figure 1:**
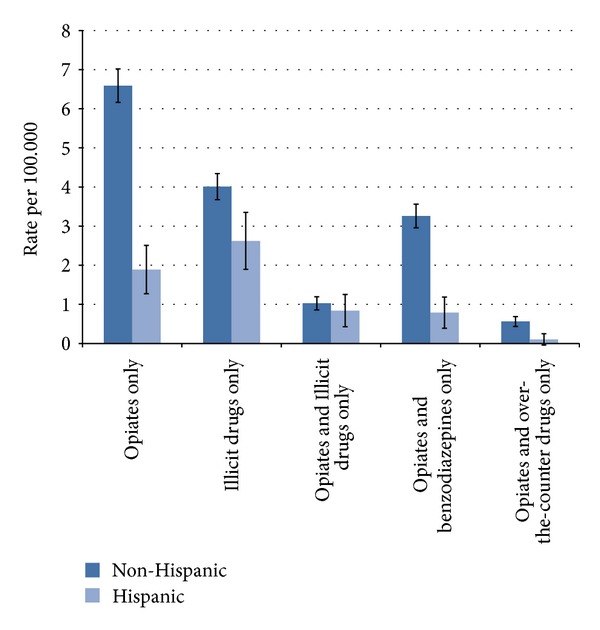
Selected combination of drug types among non-Hispanic and Hispanic decedents in Utah, 2005–2010.

Most decedents used a combination of drugs (83.7%), with the more common combinations of drug types shown for non-Hispanic and Hispanic decedents in Figure 1. Opiates only, illicit drugs only, and then opiates and benzodiazepines only were the most common in non-Hispanics, whereas illicit drugs only, opiates only, and then opiates and illicit drugs only were the most common in Hispanics. For each combination of drugs shown in Figure 1, rates were significantly greater for non-Hispanics than Hispanics (indicated by the non-overlapping 95% confidence intervals), with an exception for opiates and illicit drugs only, where there was no significant difference.

**Figure 2:**
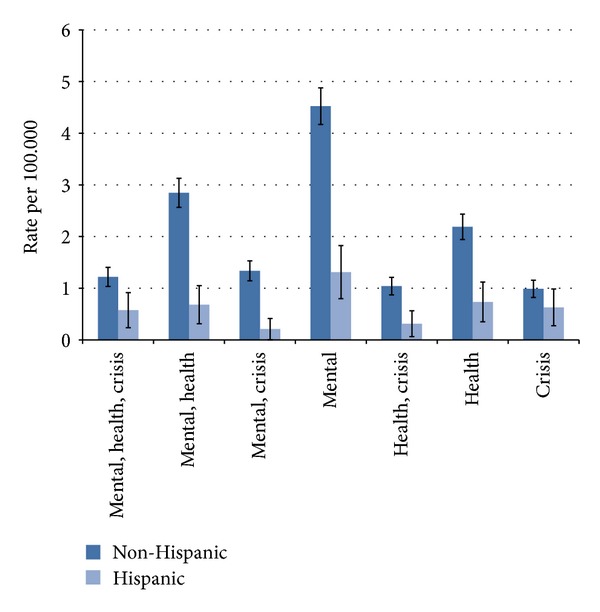
Selected combination of problems among non-Hispanic and Hispanic decedents in Utah, 2005–2010.

Selected problems were considered among the decedents (Table 3). The distribution of problems did not significantly differ between non-Hispanics and Hispanics (chi-square *P* = 0.7736). Approximately 79% of non-Hispanics and 65% of Hispanics had one or more of these problems. The most common problems were mental, physical, or crisis-related. Non-Hispanics compared with Hispanics had significantly higher rates for each of the selected problems. Combinations of mental problems, health problems, and crisis within the two weeks prior to death are presented in Figure 2. Rates of mental problems only were the greatest, then a combination of mental and physical health problems, and then physical health problems only. Rates for each combination of problems shown in Figure 2 were significantly greater in non-Hispanics than Hispanics, with the exception of crisis only.

## 4. Discussion

The results showed significantly higher rates of drug-related deaths among non-Hispanics than Hispanics from 2005 through 2010 in Utah (2.7 times for males and 2.4 times for females). A US study involving 2003–2007 data found the drug-related death rate to also be greater in non-Hispanic whites compared with Hispanics (1.4 times in males and 1.3 times in females) [11]. In 2010, a national study found the ratio of non-Hispanic whites compared with Hispanics to be 2.8 [1].

A national trend analysis of pharmaceutical opioid-related overdose deaths compared with other substance-related overdose deaths showed that from 1999 through 2009 the death rate related to pharmaceutical opioids increased fourfold and that opioids were responsible for the greatest relative increase in overdose death rates [5]. Other studies have also identified the rising rate of opioid-related deaths [2, 3, 6, 7, 9]. The number of opioid analgesic overdoses has been shown to be proportional to the amount and dose of the drug prescribed [17]. Although the current study showed that opioid use was common among both non-Hispanic and Hispanic decedents, it was less likely present in Hispanics (58.8% versus 50.0%). Another study found that in New York City, from 1990 to 2006, drug-related deaths involving analgesics were less likely in Hispanics [7].

Not only did Hispanic decedents have lower use of opiates, but they also had lower use of benzodiazepines, over-the-counter drugs, and antidepressants. A surveillance study in the United States previously showed that racial/ethnic minorities are less likely to use prescription drugs and, thus not as likely to abuse them [11]. A 2011 report showed that 40% of Hispanics compared with 10% of white non-Hispanics and 18% of other non-Hispanics in Utah did not have health insurance coverage [18]. It has also been suggested that some Hispanic immigrants lack the cultural and language fluency to navigate the healthcare system [19].

Studies indicate that the rate of drug-related deaths is greater among males than females and increases in successive age groups through 45–54 and then decreases thereafter [1, 20]. We observed this same pattern among both non-Hispanics and Hispanics in Utah. In addition, the higher drug-related death rates among non-Hispanics were seen in both males and females and across the age span. Hence, greater limitation to prescription drugs among Hispanics appears to similarly exist between both sexes and across the age groups.

Non-Hispanics had higher rates of death from not just legal drugs but illegal drugs as well. In a study covering 2002–2011 in the United States, the highest level of illegal drug use in the past month among individuals aged 12 and older was in Blacks, then Whites, then Hispanics, and lastly Asians [21]. Nevertheless, the current study found that among those who died from a drug overdose, opiates had the greatest presence in non-Hispanics and illicit drugs had the greatest presence in Hispanics, which is consistent with a lack of prescription drug access among Hispanics in the state.

Some research supports the hypothesis that Hispanics who migrate to the USA tend to have better health behaviors, are healthier, and are less likely to return to their native country [22, 23], which is consistent with our findings. For example, 37.6% of non-Hispanic compared with 31.0% of Hispanic decedents had a current physical health problem. In addition, 50.9% of non-Hispanic compared with 37.3% of Hispanic decedents had a current mental health problem. This is consistent with The First Surgeon General's Report on Mental Health, which indicated that Hispanic Americans born in the United States had similar overall rates of mental illness compared to those of Whites but that Hispanic immigrants born in Mexico or living in Puerto Rico had lower rates of mental illness than Hispanic Americans born in the United States [24].

A limitation of this study results from the inherent uncertainties of death investigations. However, as described in the Methods, several steps were taken to minimize errors in the data (e.g., extensive training of abstractors and quality checks). Second, the state of Utah has a high percentage of illegal immigrants, many of whom are of Hispanic origin who come to Utah to work [25]. Some of these individuals may not be recorded in the death records for the state. Third, ethnic misclassification may lead to an underestimation of 25–35% of Aboriginal and Hispanic drug-related deaths nationally [11]. Finally, the Utah population upon which this study is based has a comparatively high level of opiate drug and anti-depressant use and a low level of illicit drug use, which should be considered when generalizing the results.

## 5. Conclusion

The rate of drug-related death is significantly greater among non-Hispanics than Hispanics in Utah. The large difference is explained, in part, by Hispanics being less affected by the rise in prescription drug abuse, perhaps because of lower access to healthcare and prescription drugs.

The higher drug-related death rates among non-Hispanics occur in both males and females and across the age spans, indicating that greater limitations to prescription drugs among Hispanics are similar between both sexes and across the age span. Among decedents, Hispanics have a greater proportion with illegal drugs involved, consistent with their having relatively more difficult access to prescription drugs. There is some evidence that Hispanic decedents had better general physical and mental health. This may be because healthier non-native born individuals choose to migrate to the USA, are more likely to retain healthier lifestyle behaviors, and are less likely to return to their native country. It may also be that Hispanics, who have less access to healthcare, are less likely to be diagnosed with mental and physical health problems.

## Figures and Tables

**Table 1:** Sex- and age-specific drug-related deaths in Utah according to ethnicity, 2005–2010.

Demographic	Non-Hispanic				Hispanic				Rate ratio	95% CI
	Deaths	%	Rate per 100,000	95% CI	Deaths	%	Rate per 100,000	95% CI		
Sex										
Male	1,555	58.4	22.5	21.4–23.6	82	57.7	8.3	6.5–10.1	2.72	2.28–3.40
Female	1,106	41.6	16.0	15.0–16.9	60	42.3	6.6	4.9–8.2	2.44	1.88–3.16
Age										
0–24	353	13.3	6.0	5.4–6.6	23	16.2	2.3	1.3–3.2	2.64	1.73–4.02
25–34	658	24.7	30.4	28.1–32.8	40	28.2	11.5	8.0–15.1	2.64	1.92–3.63
35–44	641	24.1	39.2	36.2–42.3	40	28.2	15.5	10.7–20.2	2.54	1.85–3.50
45–54	691	26.0	42.4	39.2–45.6	28	19.7	17.9	11.3–24.6	2.37	1.62–3.45
55+	318	11.9	12.5	11.1–13.8	11	7.7	8.1	3.3–12.8	1.55	0.85–2.82

Data source: Utah Department of Health.

Note: percentages sum to 100 across rows.

**Table 2:** Specific drug types implicated in death according to ethnicity in Utah, 2005–2010.

	Non-Hispanic				Hispanic				Rate ratio	95% CI
	Deaths	%	Rate per 100,000	95% CI	Deaths	%	Rate per 100,000	95% CI		
Opiates	1,564	58.8	11.3	10.4–12.2	71	50.0	3.7	2.3–4.9	3.04	2.39–3.85
Illicit drugs	786	29.5	5.7	4.7–6.7	72	50.7	3.8	1.7–5.8	1.50	1.18–1.91
Benzodiazepines	420	15.8	3.0	2.2–3.9	20	14.1	1.0	−0.2–2.3	2.89	1.85–4.53
Over-the-counter drugs	121	7.5	0.9	0.4–1.3	5	5.6	0.3	−0.6–1.2	2.77	1.22–6.31
Antidepressants	200	4.6	1.4	1.0–1.9	8	4.2	0.4	−0.7–1.5	3.44	1.70–6.98

Data source: Utah Department of Health.

Note: percentages reflect the decedents having the selected drug type involved in their death.

**Table 3:** Selected problems among drug-related deaths according to ethnicity in Utah, 2005–2010.

	Non-Hispanic				Hispanic				Rate ratio	95% CI
	Deaths	%	Rate per 100,000	95% CI	Deaths	%	Rate per 100,000	95% CI		
Current mental health problem	1,374	50.9	9.9	9.4–10.5	53	37.3	2.8	2.0–3.5	3.57	2.71–4.70
Current physical health problem	1,010	37.6	7.3	6.8–7.7	44	31.0	2.3	1.6–3.0	3.16	2.34–4.28
Crisis in past two weeks	635	23.8	4.6	4.2–4.9	33	23.2	1.7	1.1–2.3	2.65	1.87–3.76
Alcohol problem	381	14.3	2.8	2.5–3.0	15	10.6	0.8	0.4–1.2	3.50	2.09–5.86
Recent criminal-legal problem	142	5.2	1.0	0.9–1.2	5	3.5	0.3	0.0–0.5	3.91	1.60–9.55
Job problem	102	3.7	0.7	0.6–0.9	2	1.4	0.1	0.0–0.3	7.03	1.73–28.48
Financial problem	73	2.7	0.5	0.4–0.6	3	2.1	0.2	0.0–0.3	3.35	1.06–10.64

Data source: Utah Department of Health.

Note: percentages reflect the decedents having the selected problem.
